# Investigating the Role of VDR and Megalin in Semi-Selectivity of Side-Chain Modified 19-*nor* Analogs of Vitamin D

**DOI:** 10.3390/ijms20174183

**Published:** 2019-08-26

**Authors:** Klaudia Berkowska, Aoife Corcoran, Małgorzata Grudzień, Agnieszka Jakuszak, Michał Chodyński, Andrzej Kutner, Ewa Marcinkowska

**Affiliations:** 1 Department of Biotechnology, University of Wroclaw, Joliot-Curie 14a, 50-383 Wroclaw, Poland; 2 Pharmaceutical Research Institute, Rydygiera 8, 01-793 Warszawa, Poland; 3 Department of Bioanalysis and Drug Analysis, Faculty of Pharmacy with the Laboratory Medicine Division, Medical University of Warsaw, Banacha 1, 02-097 Warszawa, Poland

**Keywords:** 1,25-dihydroxyvitamin D_3_, vitamin D analogs, differentiation, CYP24A1, vitamin D receptor, affinity, TRPV6, megalin

## Abstract

1,25-dihydroxyvitamin D_3_ (1,25D3) is implicated in many cellular functions, including cell proliferation and differentiation, thus exerting potential antitumor effects. A major limitation for therapeutic use of 1,25D3 are potent calcemic activities. Therefore, synthetic analogs of 1,25D3 for use in anticancer therapy should retain cell differentiating potential, with calcemic activity being reduced. To obtain this goal, the analogs should effectively activate transcription of genes responsible for cell differentiation, leaving the genes responsible for calcium homeostasis less active. In order to better understand this phenomenon, we selected a series of structurally related 19-*nor* analogs of 1,25D (PRI-5100, PRI-5101, PRI-5105, and PRI-5106) and tested their activities in blood cells and in cells connected to calcium homeostasis. Affinities of analogs to recombinant vitamin D receptor (VDR) protein were not correlated to their pro-differentiating activities. Moreover, the pattern of transcriptional activities of the analogs was different in cell lines originating from various vitamin D-responsive tissues. We thus hypothesized that receptors which participate in transport of the analogs to the cells might contribute to the observed differences. In order to study this hypothesis, we produced renal cells with knock-out of the megalin gene. Our results indicate that megalin has a minor effect on semi-selective activities of vitamin D analogs.

## 1. Introduction

1,25-dihydroxyvitamin D_3_ (1,25D3) is implicated in many cellular functions, including cell proliferation and differentiation, thus exerting potential antitumor effects [1]. A major limitation for therapeutic use of 1,25D3 are its potent calcemic and phosphatemic activities [2]. The method to overcome this problem is to synthesize 1,25D3 analogs with the desired anti-proliferative, pro-differentiation, and immune-modulatory effects without being hypercalcemic [3]. The understanding of the biological mechanisms through which the analogs of 1,25D3 selectively mimic anti-proliferative actions of 1,25D3, but lack its calcemic and phosphatemic activities, is scarce at present. 

1,25D3 inside its target cells binds to the specific nuclear vitamin D receptor (VDR). VDR, after binding the ligand in the cytosol, is transported to the cell nucleus, where it acts as a ligand-activated transcription factor in complex with other regulators of transcription [4]. Liganded VDR upregulates transcription of the target genes, and the gene encoding 24-hydroxylase of 1,25D3 (CYP24A1) is the most strongly upregulated one [5]. Hydroxylation of 1,25D3 at carbon atom C-24 catalyzed by CYP24A1 is the first step of its catabolism to the inactive and water soluble calcitroic acid and provides a negative feedback to the activity of 1,25D3 [6]. This makes the expression of *CYP24A1* a very useful tool to study whether VDR is functional in given cells [7]. Since the primary role of 1,25D3 is to regulate calcium homeostasis, numerous genes linked to intestinal calcium uptake are regulated by 1,25D3 [8]. One of these genes encodes a calcium channel, vanilloid transient receptor potential 6 (TRPV6), which mediates the uptake of calcium across the brush border of intestinal epithelial cells [8]. Monocyte differentiation-related genes are either regulated by 1,25D3 as primary VDR-targets or in a secondary manner. A monocytic cell differentiation marker, CD14, a co-receptor for bacterial lipopolysaccharide characteristic for monocytes and macrophages, is an example of VDR primary target [9,10]. A secondary effect of 1,25D3-induced cell differentiation is regulated among others via CCAAT-enhancer-binding protein β (C/EBPβ) transcription factor [11].

In addition, many rapid cellular responses to 1,25D3 have been described, which could not be attributed to VDR-mediated transcription, and this has led to suggestions that cells may possess other ‘non-canonical’ receptors that respond to 1,25D3 [12]. One of the most rapid cellular responses to 1,25D3 is calcium and phosphate uptake in intestinal cells, which has been attributed to the binding of 1,25D to the membrane-associated rapid response steroid-binding (MARRS) protein, also known as protein disulfide-isomerase A3 (PDIA3) [13]. Another hypothesis says that a small proportion of canonical VDR, localized to the cell membrane, might play a role in rapid intracellular signaling, through binding of 1,25D3 to an alternative ligand binding pocket of VDR [14].

The major circulating metabolite of vitamin D is 25-hydroxyvitamin D (25D), bound to the specific protein transporter, vitamin D-binding protein (DBP) [15]. 25D binds to DBP with an affinity one order of magnitude higher than 1,25D3. It has been clearly documented that transport of 25D to kidney cells is mediated via interaction of 25D bound DBP with a large transmembrane multi-ligand receptor, megalin, supported by another transmembrane receptor, cubilin [16]. Megalin is present on the surface of several endothelial cell types [17], but it has not been detected in immune cells [18]. It has been presented before that megalin also plays a role in 1,25D3 actions [19]. The same might apply to the analogs of 1,25D3.

**Figure 1:**
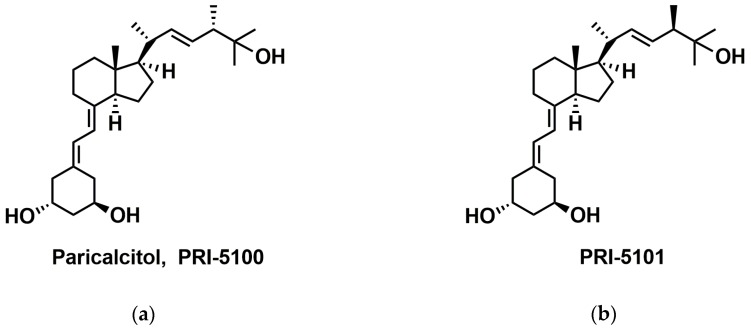

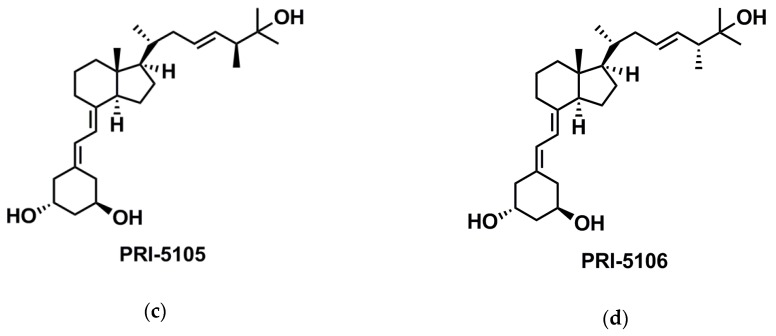
Structures of the analogs studied in this paper. (**a**) Paricalcitol, PRI-5100 (*1R,3R,7E,22E,24S*)-24-methyl-19-*nor*-9,10-secocholesta-5,7,22-triene-1,3,25-triol; (**b**) PRI-5101 (*1R,3R,7E,22E,24R*)-24-methyl-19-*nor*-9,10-secocholesta-5,7,22-triene-1,3,25-triol; (**c**) PRI-5105 (*1R,3R,7E,22E,24S*)-24-methyl-19-*nor*-20a-homo-9,10-secocholesta-5,7,22-triene-1,3,25-triol; (**d**) PRI-5106 (*1R,3R,7E,22E,24R*)-24-methyl-19-*nor*-20a-homo-9,10-secocholesta-5,7,22-triene-1,3,25-triol.

Out of a broad collection of our vitamin D analogs, we selected for these studies a panel of analogs of 1,25-dihydroxyvitamin D_2_ (1,25D2), a metabolite of plant vitamin D form [20]. Our structurally related 19-*nor* analogs have either a single or a double structural modification and a gradually increased biological activity. PRI-5100 (paricalcitol) is a 19-*nor* analog of 1,25D2_._ PRI-5101 differs from PRI-5100 only in the absolute configuration at C-24 in the side-chain. The 19-*nor* analogs, PRI-5105 and PRI-5106, are additionally modified in the side chain and are the homologues of PRI-5100 and PRI-5101, respectively (Figure 1). The whole series of analogs were shown to be less calcemic than 1,25D3 [21,22]. Therefore, we used this series to understand how these analogs are able to split their calcemic and differentiation-inducing actions by studying their activity in blood, intestinal, bone, and in kidney cells.

## 2. Results

### 2.1. Binding of Analogs to VDR

1,25D3 and its analogs, in order to activate transcription of their target genes, must bind to VDR’s ligand binding domain. Thus, the affinities of 1,25D3 and analogs PRI-5100, PRI-5101, PRI-5105, and PRI-5106 to recombinant VDR protein were tested. The experiments were performed using a fluorescence polarization (FP)-based competition assay. The binding of analogs to VDR was tested in a wide range of concentrations, and was compared to the binding of 1,25D3 to the receptor. Dose– response curves were plotted, and IC_50_ values were calculated from these dose–response curves and presented in Table 1.

### 2.2. Biological Actions of Analogs in Acute Myeloid Leukemia Cells

The potential of analogs to induce monocytic differentiation was screened in acute myeloid leukemia HL60 cells exposed to 1,25D3 and analogs in a wide range of concentrations. CD14 is a monocyte cell surface marker, which is transcriptionally regulated by 1,25D3 in a primary manner by VDR [10] and in a secondary manner by C/EBPβ transcription factor [23] upregulated by 1,25D3 [11]. The expression of CD14 was tested in flow cytometry in HL60 cells exposed to 1,25D3 or to analogs for 96 hours and dose–response curves were plotted. EC_50_ values were calculated for 1,25D3 and each of the analogs tested. These values are presented in Table 2.

**Figure 2:**
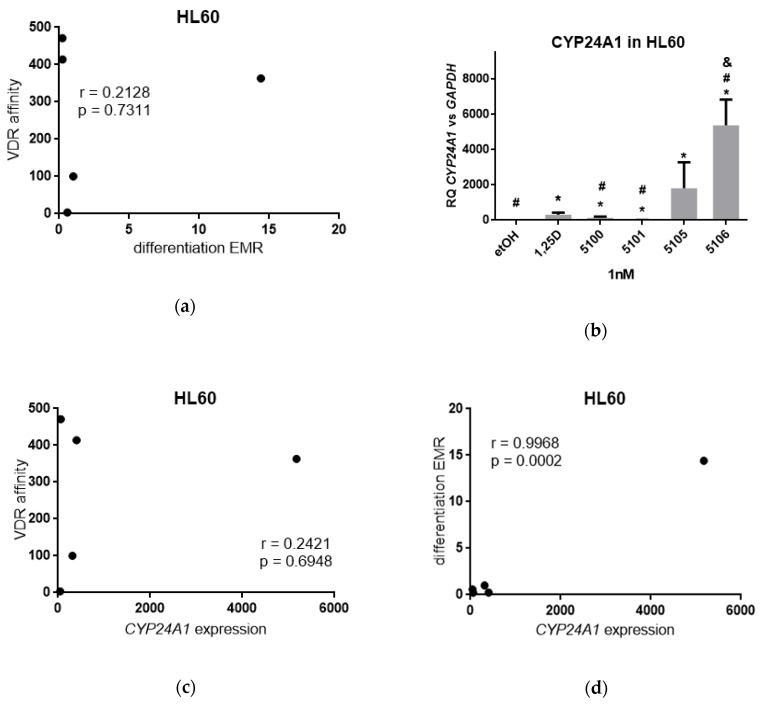
Activities of analogs towards HL60 cells. (**a**) Correlation between affinities of the analogs to VDR from Table 1 and their differentiation-inducing activities from Table 2; (**b**) HL60 cells were exposed to 1 nM 1,25D3, or 1 nM analogs and after 96 h the expression of *CYP24A1* mRNA was measured by real-time PCR. The bar charts show the mean values (±SD) of the fold changes in mRNA levels relative to *GAPDH* mRNA levels. Values that differ significantly from those obtained for control cells are marked with *, while the values that differ significantly from those obtained for 1,25D3-treated cells are marked by #. The value significantly higher than that induced by PRI-5105 is marked with &; (**c**) Correlation between affinities of the analogs to VDR from Table 1 and their *CYP24A1*-inducing activities; (**d**) Correlation between differentiation-inducing activities from Table 2 and their *CYP24A1*-inducing activities.

Then, the correlation between affinities to VDR (RBA values) and effective molar ratio (EMR) values for respective analogs were plotted and are presented in Figure 2a. The graph indicates that there is no correlation between these two values, which suggests that binding of a given analog to VDR is not the most important factor responsible for biological effects towards HL60 cells. In our next experiments, we tested expression of *CYP24A1* in HL60 cells exposed to 1,25D3 or to the analogs. From our previous experiments, we know that the kinetics of 1,25D3-induced *CYP24A1* expression is slow [24], therefore we exposed HL60 cells to analogs for 96 hours. On the other hand, HL60 cells are very sensitive to 1,25D3, thus we applied the compounds at 1 nM concentration. The results obtained in real-time PCR are presented in Figure 2b. All compounds tested significantly upregulated expression of *CYP24A1*, when compared to control (exposed to solvent) sample (marked with *). However, the expression of *CYP24A1* induced by PRI-5106 was significantly higher than that induced by 1,25D3 (marked with #), and significantly higher than that induced by PRI-5105 (marked with &). Then, the values of *CYP24A1* expression induced by tested compounds were plotted against affinities to VDR (RBA values), and again no correlation was found (Figure 2c). However, differentiation of HL60 cells induced by tested compounds strictly correlated with the *CYP24A1* expression they induced (Figure 2d).

### 2.3. Biological Actions of Analogs in Intestine Cells

**Figure 3:**
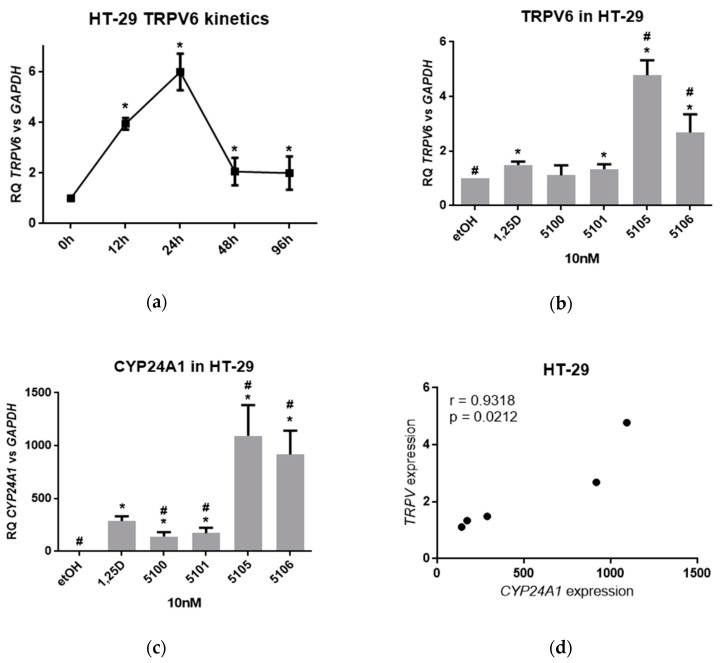
Activities of analogs towards HT-29 cells. (**a**) HT-29 cells were exposed to 10 nM 1,25D3 for a different time and then the expression of *TRPV1* mRNA was measured by real-time PCR. The graph shows mean values (±SD) of the fold changes in mRNA levels relative to *GAPDH* mRNA levels. Values that differ significantly from those obtained for control cells are marked with *; (**b**) HT-29 cells were exposed to 10 nM 1,25D3, or 10 nM analogs and after 24 h the expression of *TRPV1* mRNA was measured by real-time PCR. The bar charts show the mean values (±SD) of the fold changes in mRNA levels relative to *GAPDH* mRNA levels. Values that differ significantly from those obtained for control cells are marked with *, while the values that differ significantly from those obtained for 1,25D3-treated cells are marked by #; (**c**) HT-29 cells were exposed to 10 nM 1,25D3 or 10 nM analogs, and after 24 h the expression of *CYP24A1* mRNA was measured by real-time PCR. The graph shows the mean values (±SD) of the fold changes in mRNA levels relative to *GAPDH* mRNA levels. Values that differ significantly from those obtained for control cells are marked with *; (**d**) Correlation between *TRPV*-inducing activities of analogs and their *CYP24A1*-inducing activities.

Intestine is a tissue important for calcium and phosphate absorption. TRPV6, which is located in the brush border membranes of the intestinal cells, is a potential mediator of calcium uptake from the diet [25]. The gene encoding this calcium channel is directly regulated by 1,25D3 [8] by activating multiple vitamin D receptor binding sites [26]. Therefore, we hypothesized that 1,25D3 and its analogs might regulate this gene with a variable strength. To investigate this, we first determined the kinetics of 1,25D3-induced *TRPV6* gene upregulation in HT-29 cells. Figure 3a shows that *TRPV6* gene upregulation in response to 1,25D3 is rather fast in HT-29 cells, with a maximum at 24 hours from exposure. Then, HT-29 cells were exposed to either 1,25D3 or to the analogs at 10 nM concentrations for 24 hours and expressions of *TRPV6* and *CYP24A1* were tested in real-time PCR. The results are presented in Figure 3b,c, respectively, and they show that *CYP24A1* is upregulated in HT-29 cells to the higher fold than *TRPV6*, but the pattern of upregulation in response to studied compounds is similar. In HT-29 cells, the analog PRI-5105 induced the strongest expression of target genes, and PRI-5106 was slightly less effective (difference not significant). PRI-5105 and PRI-5106 upregulated both genes expression significantly stronger than 1,25D3. The visible similarity between graphs presented in Figure 3b,c was further confirmed by a correlation coefficient presented in Figure 3d. The data presented in Figure 2 and Figure 3 confirm that expression of *CYP24A1* induced by vitamin D analogs is a very good marker of their activities.

### 2.4. Biological Actions of Analogs in Bone and Kidney Cells

**Figure 4:**
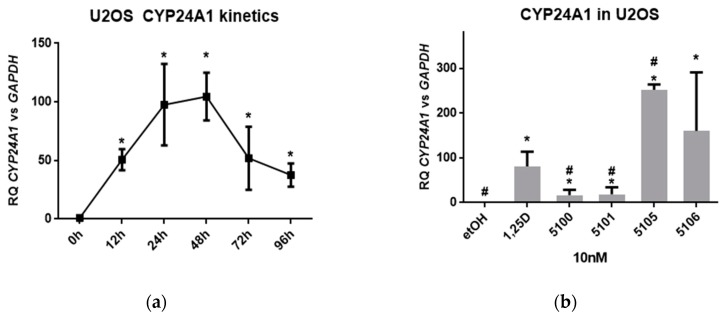

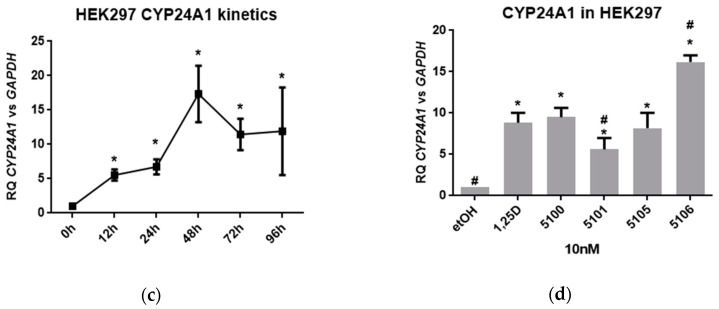
Activities of analogs towards U2OS and HEK297 cells. (**a**) U2OS cells were exposed to 10 nM 1,25D3 for different times and then the expression of *CYP24A1* mRNA was measured by real-time PCR. The graph shows mean values (±SD) of the fold changes in mRNA levels relative to *GAPDH* mRNA levels. Values that differ significantly from those obtained for control cells are marked with *; (**b**) U2OS cells were exposed to 10 nM 1,25D3, or 10 nM analogs, and after 48 h the expression of *CYP24A1* mRNA was measured by real-time PCR. The bar charts show the mean values (±SD) of the fold changes in mRNA levels relative to *GAPDH* mRNA levels. Values that differ significantly from those obtained for control cells are marked with *, while the values that differ significantly from those obtained for 1,25D3-treated cells are marked by #; (**c**) HEK297 cells were exposed to 10 nM 1,25D3 for different time and then the expression of *CYP24A1* mRNA was measured by real-time PCR. The graph shows mean values (±SD) of the fold changes in mRNA levels relative to *GAPDH* mRNA levels. Values that differ significantly from those obtained for control cells are marked with *; (**d**) HEK297 cells were exposed to 10 nM 1,25D3, or 10 nM analogs and after 48 h the expression of *CYP24A1* mRNA was measured by real-time PCR. The bar charts show the mean values (±SD) of the fold changes in mRNA levels relative to *GAPDH* mRNA levels. Values that differ significantly from those obtained for control cells are marked with *, while the values that differ significantly from those obtained for 1,25D3-treated cells are marked by #.

The next cell type examined was bone. All types of bone cells are regulated in response to 1,25D3. 1,25D3 promotes differentiation of osteoprogenitors to mature osteoblasts, and also participates in the activation of osteoclasts important for bone remodeling [2,27]. We used U2OS cells as a model of osteoblast-derived cells. First, the kinetics of 1,25D3-induced *CYP24A1* gene upregulation in U2OS cells was tested. The maximum upregulation of *CYP24A1* expression was observed at 48 hours from the exposure to 10 nM 1,25D3 (Figure 4a). Therefore, in the next series of experiments, U2OS cells were exposed to 10 nM analogs for 48 hours, and expression of *CYP24A1* was tested. As presented in Figure 4b, the pattern of analog-induced *CYP24A1* expression in U2OS cells was different from that observed in HL60 cells, but resembled the one observed in HT-29 cells. In HL60 cells, the analog PRI-5106 was significantly more potent than any of the other compounds tested. In HT-29 and in U2OS cells, the most potent analog was PRI-5105.

The last type of cells tested in our experiments were kidney cells. Kidneys are the central organs for vitamin D metabolism and for calcium homeostasis [28]. Figure 4c shows that maximum *CYP24A1* gene upregulation in HEK297 cells in response to 1,25D3 is at 48 hours from exposure. Therefore, next, the HEK297 cells were exposed to 10 nM analogs for 48 hours, and expression of *CYP24A1* was tested. As presented in Figure 4d, the pattern of analog-induced *CYP24A1* expression in HEK297 cells was different from that observed in any other cells examined. In HEK297 cells, only the analog PRI-5101 activated expression of *CYP24A1* less than 1,25D3. The highest expression was induced by PRI-5106. It is noteworthy that in HEK297 cells, the expression of *CYP24A1* was upregulated, at most, several times, while in U2OS cells the expression was upregulated hundreds of times.

### 2.5. Expression of Megalin in Tested Cells

**Figure 5:**
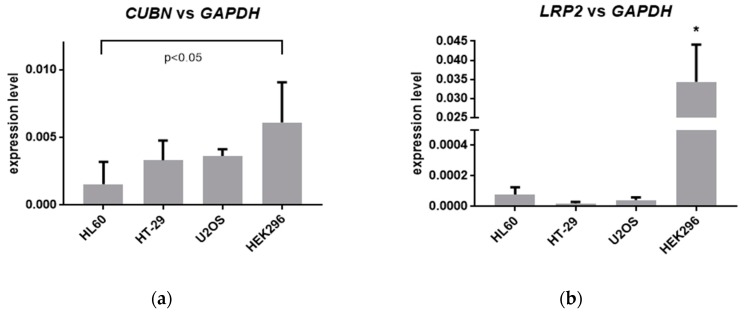
Expression of genes encoding cubilin and megalin in all cells tested. (**a**) The bar charts show the mean values (±SD) of the expression of *CUBN* mRNA, relative to *GAPDH* mRNA levels in all cells tested; (**b**) The bar charts show the mean values (±SD) of the expression of *LRP2* mRNA, relative to *GAPDH* mRNA levels in all cells tested. Values that differ significantly from the respective value obtained for HL60 cells are marked with *.

Since in vitro activities of tested analogs were different in different cell types, we hypothesized that this effect might be due to the variable effectiveness of transport of these analogs into the cells tested. The receptors which have been known to facilitate transport of vitamin D compounds to the cells are megalin and cubilin. Thus, in the next step, the expression of genes encoding these receptors in HL60, HT-29, U2OS, and HEK297 cells were examined. The results obtained (Figure 5) indicate that the expression of *CUBN*, the gene encoding cubilin is low in all cell lines tested, with the highest expression level in HEK297 cells. The expression of *LRP2* gene, which encodes megalin, was much more differentiated between cells from different tissues. The expression of *LRP2* is high in kidney cells, and very low in HL60 and U2OS, while in HT-29 it is barely detectable.

### 2.6. Knock-Out of Megalin in HEK297 Cells

**Figure 6:**
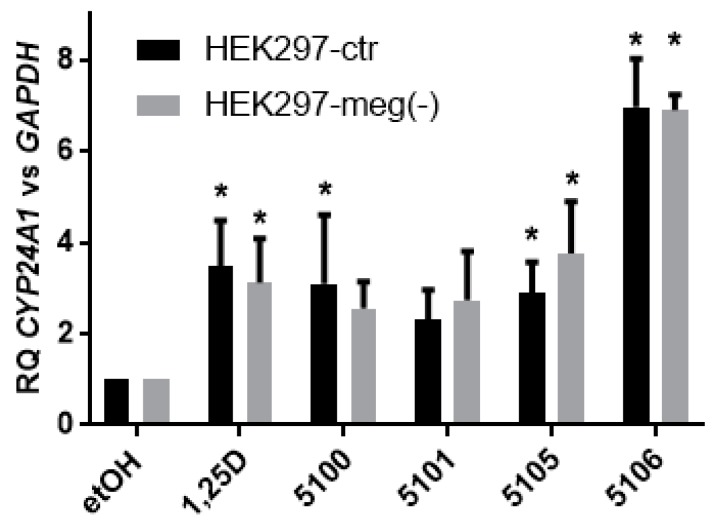
Expression of *CYP24A1* in response to 1,25D3 and to analogs in HEK297-ctr and HEK297-meg(−) cells. The cells were exposed to 10 nM 1,25D3 or 10 nM analogs, and after 48 h the expression of *CYP24A1* mRNA was measured by real-time PCR. The bar charts show the mean values (±SD) of the fold changes in mRNA levels relative to *GAPDH* mRNA levels. Values that differ significantly from those obtained for respective control cells are marked with *.

Based on the above experiments, we decided to knock-out the LRP2 gene in HEK297 cells, in which its expression was the highest. For that purpose, clustered regularly interspaced short palindromic repeats (CRISPR) and CRISPR-associated protein (Cas9) with double nickase plasmid procedure was used. Using this procedure, two sublines of HEK297 cells were generated: HEK297-ctr with wild type megalin, and HEK297-meg(−), with homozygous deletion in the LRP2 gene. These HEK297 sublines were exposed to 10 nM analogs for 48 hours, and expression of CYP24A1 was tested. The results of quantification of CYP24A1 relative to GAPDH expression are presented in Figure 6.

The results indicate that both genetical modifications and/or culture of the cells in puromycin containing medium have caused the cells to become less responsive to 1,25D3 or to the analogs than the wild type HEK297 cells. However, the pattern of the activities of the compounds remained similar to that in wild type cells (compare to Figure 4d), except that in HEK297-meg(−), cells activity of PRI-5100 was significantly lower than activities of all other compounds.

## 3. Discussion

The mechanisms of semi-selective biological activities of vitamin D analogs have been debated for many years, however, they are still poorly understood [3,29,30]. Thousands of analogs have been synthesized, and many of them are semi-selective in their biological actions [31]. For example, some analogs which are very effective in inducing cell differentiation exert low calcemic activities [21]. Most probably, the mechanisms that split different biological actions of 1,25D3 in analogs consist in complex interplay between proteins which interact with either 1,25D3 or analogs [30]. The following proteins have been proposed to participate in this interplay: the nuclear VDR, the serum DBP, CYP24A1, and other metabolizing enzymes, the membrane receptors that mediate the rapid actions of vitamin D compounds and intracellular binding proteins that can facilitate both metabolism and VDR activation [30]. Moreover, certain non-calcemic metabolites of 1,25D3, which are produced in human skin, activate or inhibit nuclear receptors other than VDR. These are either orphan nuclear receptors RORα and RORγ [32], or aryl hydrocarbon receptor (AhR) [33]. Whether or not our analogs bind to the above-mentioned nuclear receptors has not been yet determined.

Here, we hypothesized that megalin, a protein which facilitates transport of DBP-bound vitamin D to kidney cells and takes part in the actions of 1,25D3, may participate in selective actions of vitamin D analogs.

In our paper, we describe a series of vitamin D analogs which exert various patterns of induction of *CYP24A1* expression in cells of various origins. In particular, the activity of PRI-5106 was significantly higher than of the other compounds studied in HL60 cells. In contrast, in HT-29 and U2OS cells, PRI-5105 was the most active one in our experiments. Moreover, in HEK297 cells, expression of *CYP24A1* was upregulated in response to 10 nM compounds, at most, several times, while in U2OS, cells were upregulated hundreds of times. In HT-29, the upregulation was higher than one thousand times, and in HL60 cells it reached a few thousand in response to 1 nM compounds. These differences might be caused by variable transcriptional activities of VDR in these cells, but also by different background levels of *CYP24A1* expression.

It has been known in the field since the early nineties that extending the side-chain of vitamin D by one or two carbons usually increases the cell differentiating activity [24,34]. However, extension of the natural side-chain of a 19-*nor* analog of 1,25-dihydroxyvitamin D_2_ (PRI-5100) by one carbon (C-20a) resulted in a loss of its VDR affinity. The binding affinity of PRI-5105 dropped about one hundred times compared to the parent PRI-5100. This loss of binding affinity was not accompanied by the reduction of the potency of the analog to induce monocytic differentiation in HL60 cells. This potency was higher for the side-chain extended PRI-5105 than for the parent PRI-5100, although still being low for both. On the other hand, PRI-5106, with the same extension of the side-chain and the synthetic unnatural 24*R* configuration, retained its VDR affinity and, very beneficially, showed over sixty times increased potency in inducing monocytic differentiation of HL60 cells. This potency was fifteen times higher for our synthetic PRI-5106 than that of the natural 1,25D3. Additionally, the expression of *CYP24A1* induced by PRI-5106 was significantly higher than that of PRI-5105. PRI-5106 was also the most potent one towards kidney HEK297 cells. Therefore, it is of great interest to optimize the structure of PRI-5106 further to increase its potency. The optimization should be rather focused on the A-ring, leaving the synthetic side-chain of PRI-5106 untouched or further extended by additional carbon (20b). In intestinal HT-29 and in osteoblast-derived U2OS cells, the relative activities of PRI-5105 and PRI-5106 were reversed. It was PRI-5105 that induced the strongest expression of target genes. In HT-29 cells, both PRI-5105 and PRI-5106 upregulated expression of *TRPV6* and *CYP24A1* genes significantly stronger than 1,25D3. Nevertheless, lead analog is to be developed and optimized almost for each cell type, separately.

Next, we wondered which proteins could mediate these variable activities. As we presented above, the binding of the analogs to VDR neither correlated to the induction of cell differentiation nor to the induction of expression of *CYP24A1*. The activity of metabolizing enzymes should also not be important for short term effects in vitro. Therefore, we decided to address membrane receptors responsible for the transportation of vitamin D. We tested the expression of cubilin and megalin in our cell lines. The expression of megalin encoding gene, *LRP2*, appeared to be very diverse in different cells. The highest expression of *LRP2* was in kidney cells, and these cells were selected for knock-out experiments. In the next step, we generated two sub-lines of HEK297 cells, one transfected with control plasmid, and one with homozygous deletion in *LRP2* gene. Our experiments revealed that both genetically modified cell lines responded to 1,25D3 and to analogs to a lower extent than wild type HEK297 cells. On the other hand, the activity of the compounds tested was similar in both sublines of HEK297 cells, which suggests that megalin has a minor role in the semi-selectivity of vitamin D analogs.

## 4. Materials and Methods

### 4.1. Cell Lines

HL60, HT-29, and HEK297-FRT (HEK297) cells were from the institute of immunology and experimental therapy in Wroclaw (Poland). U2OS were purchased from the German resource center for biological material (DSMZ GmbH, Braunschweig, Germany). HL60 cells were propagated as suspension cultures in RPMI 1640, while HT-29, U2OS, and HEK297 adherent cultures in DMEM medium supplemented with 10% fetal calf serum (FCS, Sigma, St. Louis, MO, USA), 100 units/mL penicillin, and 100 µg/mL streptomycin (Sigma, St. Louis, MO, USA). The cells were kept at standard cell culture conditions, i.e. humidified atmosphere of 95% air and 5% CO_2_ at 37 °C. The cell number and viability were determined by hemocytometer counts and trypan blue (0.4%) exclusion. For all experiments, the cells were suspended in fresh medium containing 1,25D3, analog, or the equivalent volume of ethanol as a vehicle control.

### 4.2. Chemicals and Antibodies

1,25D3 and all analogs were synthesized in the pharmaceutical research institute (Warsaw, Poland). The compounds were aliquoted and stored in glass ampoules under argon at −20°C. Compounds were dissolved in an absolute ethanol to 100 µM, and subsequently diluted in the culture medium to the required concentration. Antibodies CD14-PE and isotype control-PE were from ImmunoTools (Friesoythe, Germany).

### 4.3. Determination of Cell Differentiation

The expression of CD14 was determined by flow cytometry. The cells were incubated with 1,25D3 or analogs and then stained with 1 µL of CD14-PE for 1 hour on ice. Next, they were washed three times with ice-cold PBS and suspended in 0.5 ml PBS prior to analysis on FACS Calibur flow cytometer (Becton Dickinson, San Jose, CA, USA). The acquisition parameters were set for an isotype control. Data analysis was performed with use of WinMDI 2.8 software (freeware by Joseph Trotter).

### 4.4. cDNA Synthesis and RealTime PCR

Total RNA was isolated using Tri Reagent (Sigma) as per manufacturer’s recommendations. RNA quantity was determined using Nanodrop (Thermo Fisher Scientific Inc. Worcester, MA, USA) and the quality of RNA was verified by gel electrophoresis. RNA was transcribed into cDNA using high capacity cDNA reverse transcription kit (Applied Biosystems, Foster City, CA, USA). Real-time RT-PCR reaction was performed using SensiFAST SYBR Hi-ROX 2000 (Bioline, London, UK) and CFX real-time PCR System (Bio-Rad Laboratories Inc., CA, USA). The sequences of *CYP24A1* and *GAPDH* primers and reaction conditions were as described previously [24]. The sequences of *TRPV6*, *LRP2*, and *CUBN* primers were:

*LRP2*: 5′-GCT CTC GTC GCC TGC CTA; 5′-ACA ACA GCG CAG CCA ATT TCA;

*CUBN*: 5′-TCT TCC AGT CTC AGG AGG CAC; 5′-ACA GCG GAA CGA GCT TCT AAG TG;

Quantification of gene expression was analyzed with either the ΔCq or ΔΔCq method using *GAPDH* as the endogenous control. Primer’s efficiencies were measured in all cell lines using a real-time PCR reaction based on the slope of the standard curve. The results were normalized to primer efficiencies to compare gene expression in different cell lines. Real-time PCR assays were performed at least in triplicate.

### 4.5. Human VDR Binding Assay

Binding affinity to VDR was evaluated using a Polarscreen Vitamin D receptor competitor assay, Red (Life Technologies, Carlsbad, CA, USA), under manufacturer’s conditions. The polarized fluorescence was measured using Envision (Perkin-Elmer, Waltham, MA, USA). All compounds were evaluated within the range 10^−11^ to 10^−6^ M. IC_50_ values were calculated using the average of measured values using GraphPad Prism 7 (GraphPad Software, San Diego, CA, USA).

### 4.6. Knock-Out of LRP2 Gene Using CRISPR/Cas9 Method

*LRP2* was knocked out using the megalin double nickase plasmid (h2) (sc-401094-NIC-2), while control cells were generated using control double nickase plasmid (sc-437281) (both from Santa Cruz Biotechnology Inc. (Santa Cruz, CA, USA), according to the manufacturer’s protocol. In brief, HEK293 were seeded onto 6-well plates and after two days in culture, 1 μg/well of respective plasmid suspended in UltraCruz® Transfection Reagent (Santa Cruz: sc-395739) was added to the wells. After 48 h, puromycin (1 μg/mL) was added to the cell culture. The clones of transfected cells were selected using single cell dilution protocol. After 14 days in culture, the colonies of the cells growing from single cells were harvested and screened for mutations in the *LRP2* gene.

The following pairs of primers were used to test the presence of mutation in selected clones:

Pair 1: 5′-TCACACCACTCTGGAATAACTG; 5′-GCACTAAGGCAGGACACTC;

Pair 2: 5′-TCACACCACTCTGGAATAACTG; 5′-TTCACTTGGGATACACTGACC;

Pair 3: 5′-TGAATACAGGTGCGACCAC; 5′-GACAAGGGTGAAACCAAATACAG.

Sequencing was performed by Microsynth AG (Balgach, Switzerland) and analyzed using DSDecodeM [35]. Based on the sequencing, two clones were selected for further experiments. Clone 1C50B2 transfected using control double nickase plasmid with wild-type megalin (HEK293-ctr) and clone 2M2A10 (HEK293-meg(−)) with homozygous deletion in *LRP2* gene.

### 4.7. Statistical Analysis

All experiments were repeated at least three times. The Student’s *t*-test for independent samples was used to compare two groups of results, and two-way ANOVA was used to compare results obtained for control and knock-out cells (GraphPad Prism 7; GraphPad Software Inc. San Diego, CA, USA).

## 5. Conclusions

The anticancer and calcemic actions of 1,25D3 analogs are separable, however the mechanism of separation has not been fully understood. In our paper, we present a series of vitamin analogs, which not only are less calcemic than 1,25D3, but also have different patterns of activities in cells from different tissues. We hypothesized that megalin, which possibly participates in the transport of the analogs to the kidney cells, might contribute to the observed differences. Experiments performed in order to test our hypothesis showed that megalin has a minor role in the semi-selectivity of vitamin D analogs.

## Figures and Tables

**Table 1:** Affinities of the compounds to recombinant VDR.

	1,25D3	PRI-5100	PRI-5101	PRI-5105	PRI-5106
**IC_50_ (M)**	2.320 × 10^−9^	5.599 × 10^−10^	4.921 × 10^−10^	6.336 × 10^−8^	6.389 × 10^−10^
**RBA ^a^**	100	414	471	3.66	363

The VDR binding affinity is expressed as IC_50_ and relative binding affinity (RBA). ^a^ The potency of 1,25D3 is normalized to 100.

**Table 2:** Monocytic differentiation induced by 1,25D3 and analogs.

	1,25D3	PRI-5100	PRI-5101	PRI-5105	PRI-5106
**EC_50_ (M)**	2.745 × 10^−10^	1.128 × 10^−9^	1.179 × 10^−9^	4.763 × 10^−10^	1.901 × 10^−11^
**EMR**	1	0.24	0.23	0.58	14.4

Cell differentiation activity of compounds is expressed as EC_50_: the concentration which induces half-maximal expression of CD14 cell surface antigen in HL60 cells and EMR: effective molar ratio (EC_50_ 1,25D3/EC_50_ analog).

## Abbreviations

1,25D3	1,25-dihydroxyvitamin D_3_
AhR	aryl hydrocarbon receptor
C/EBPβ	CCAAT-enhancer-binding protein β
CYP24A1	24-hydroxylase of 1,25D3
DBP	vitamin D-binding protein
EMR	effective molar ratio
FP	fluorescence polarization
MARRS	membrane associated rapid response steroid-binding protein
RBA	relative binding affinity
TRPV6	vanilloid transient receptor potential 6
VDR	vitamin D receptor
